# Algorithms to prescribe Schroth exercises for each of four Schroth curve types

**DOI:** 10.1186/1748-7161-7-S1-P22

**Published:** 2012-01-27

**Authors:** EM Watkins, S Bosnjak, EC Parent

**Affiliations:** 1 University of Alberta, Faculty of Rehabilitation Medicine Edmonton, Canada; 2 University of Alberta/Alberta Health Services Edmonton, Canada

## Background

Systematic reviews have shown that most exercise studies for scoliosis treatment lacked standardization of exercise prescription. Schroth exercise prescription is based on scoliosis curve type with specific exercises designed to target different aspects of the spinal curve and different areas of the body. The intensity of exercises is increased based on patient capacity. There may be dose dependant and exercise specific effects, therefore it is important to adopt a standardized method of prescription, especially in clinical research trials.

Goal: To describe prescription algorithms and a performance checklist for standardizing Schroth exercise treatment based on instructions in the Schroth training.

## Materials and methods

Prescription algorithms to guide progression in intensity and from isometric to dynamic exercises were developed by two Schroth-certified therapist-researchers and a physiotherapy professor. Intensity increases by dosage and by exercise type - from gravity assisted postural shifts to active postural shifts against gravity. The performance checklist was developed to ensure adequate exercise performance based on key Schroth principles of breathing and autocorrection.

## Results

An exercise prescription algorithm has been designed for each of the four Schroth curve types. The patient begins with the “Sitting-on-a-ball” exercise. If performance assessed using the proposed checklist is adequate, the next exercise in the algorithm is attempted. Otherwise, the patient attempts the easier exercise. Adequate performance at start intensity as rated by the checklist, leads to dosage increase to target intensity.

## Conclusions

The proposed algorithms and performance checklist will be used to standardize exercise prescription in a randomized control trial.

